# Combination of gene set signatures correlates with response to nivolumab in platinum-resistant ovarian cancer

**DOI:** 10.1038/s41598-021-91012-w

**Published:** 2021-06-01

**Authors:** Ryusuke Murakami, Junzo Hamanishi, J. B. Brown, Kaoru Abiko, Koji Yamanoi, Mana Taki, Yuko Hosoe, Ken Yamaguchi, Tsukasa Baba, Noriomi Matsumura, Ikuo Konishi, Masaki Mandai

**Affiliations:** 1 Department of Gynecology and Obstetrics, Kyoto University Graduate School of Medicine, 54 Kawahara-cho, Shogoin, Sakyo-ku, Kyoto 606-8501 Japan; 2 Department of Gynecology, Shiga General Hospital, 5-4-30, Moriyama, Moriyama-city, Shiga 524-8524 Japan; 3 Laboratory for Molecular Biosciences, Life Science Informatics Research Unit, Kyoto University Graduate School of Medicine, Yoshida-Konoe-cho, Sakyo-ku, Kyoto 606-8501 Japan; 4 Center for Cancer Immunotherapy and Immunobiology, Kyoto University Graduate School of Medicine, 54 Kawahara-cho, Shogoin, Sakyo-ku, Kyoto 606-8501 Japan; 5 Department of Obstetrics and Gynecology, National Hospital Organization Kyoto Medical Center, 1-1 Fukakusa Mukaihata-cho, Fushimi-ku, Kyoto 612-8555 Japan; 6 Department of Obstetrics and Gynecology, Faculty of Medicine, Iwate Medical University, 19-1, Uchimaru, Morioka, Iwate 028-3694 Japan; 7 Department of Obstetrics and Gynecology, Faculty of Medicine, Kindai University, 377-2, Ohnohigashi, Osakasayama, Osaka 589-0014 Japan

**Keywords:** Ovarian cancer, Cancer immunotherapy

## Abstract

Based on our previous phase II clinical trial of anti-programmed death-1 (PD-1) antibody nivolumab for platinum-resistant ovarian cancer (n = 19, UMIN000005714), we aimed to identify the biomarkers predictive of response. Tumor gene expression was evaluated by proliferative, mesenchymal, differentiated, and immunoreactive gene signatures derived from high-grade serous carcinomas and a signature established prior for ovarian clear cell carcinoma. Resulting signature scores were statistically assessed with both univariate and multivariate approaches for correlation to clinical response. Analyses were performed to identify pathways differentially expressed by either the complete response (CR) or progressive disease (PD) patient groups. The clear cell gene signature was scored significantly higher in the CR group, and the proliferative gene signature had significantly higher scores in the PD group where nivolumab was not effective (respective *p *values 0.005 and 0.026). Combinations of gene signatures improved correlation with response, where a visual projection of immunoreactive, proliferative, and clear cell signatures differentiated clinical response. An applicable clinical response prediction formula was derived. Ovarian cancer-specific gene signatures and related pathway scores provide a robust preliminary indicator for ovarian cancer patients prior to anti-PD-1 therapy decisions.

## Introduction

Ovarian cancer, the leading cause of death among gynecological malignancies, includes high-grade serous (75%), endometrioid (10%), clear cell (10%), and mucinous (3%) carcinomas in histopathological subtyping^1^. Standard first-line chemotherapy employs a combination of taxane and platinum agents. Clear cell ovarian carcinoma, with an increased prevalence in East Asian populations, is notably resistant to chemotherapy^2,3^.

Many cancer types have been studied in depth to identify transcriptome profiles in bulk tumor tissue that correlate with response to chemotherapy. In ovarian cancer, we have previously shown that the correlation between tumor microenvironment and subtype-specific transcriptome profiles, resulting in the “Classification of Ovarian Cancer” (CLOVAR) gene expression signatures^4^, could be indicative of antitumor and prognostic response to individual small molecule chemotherapeutic agents^5^. Particularly, the ovarian mesenchymal subtype has the worst prognosis but is responsive to taxane^6,7^.

Gene expression continues to be a central focal point as cancer therapy strategies broaden to incorporate the emerging use of immune checkpoint inhibitors that target the immunosuppressive resistance mechanisms of tumors^8^.

In a recent study, Liu et al. reported the results of a phase II study (n = 38) combining the immune checkpoint anti-programmed death-1 (PD)-1 inhibitor nivolumab and bevacizumab in relapsed ovarian cancer. In this study, patients were stratified according to tumor resistance or sensitivity to platinum^9^, and the findings showed that platinum-sensitive patients responded better than platinum-resistant patients. Concurrently, we previously executed a 19-patient phase II study of nivolumab exclusively for platinum-resistant recurrent ovarian cancer. In this study, two patients, including one patient with clear cell carcinoma, demonstrated a complete response (CR) to the therapy, contributing to an overall response rate of 15% and a disease control rate of 45%^10^. Although tumor expression of the programmed death-1 ligand 1 (PD-L1) is predictive of the therapeutic response in certain tumor types such as melanoma and non-small cell lung cancer^11–15^, we found that PD-L1 expression alone was not sufficiently predictive of response in the case of ovarian cancer^10,16^.

Despite reports that immune checkpoint inhibitor therapy is demonstrating remarkable and durable response in a number of solid and hematological cancers^17–21^, response rates are still far from optimal^9^, and therefore, there is a pressing need to develop appropriate predictive biomarkers^22^. This is notable for ovarian cancer, where regulatory approval for clinical therapy by immune checkpoint inhibitors has not occurred in any country^23^.

Although gene-specific expression can be used to determine consistent expression-response correlations, it is also possible to consider tumor- and immune-contextualized functional groups of gene expression and their correlations with specific outcomes^24,25^. This is important when considering that ovarian cancer has been demonstrated to be difficult to analyze solely by individual transcript levels^10,16^ and is compounded by the fact that heterogeneous bulk tissue RNA analysis is still the most practical strategy for clinical settings. Furthermore, given that the combination of small molecule and anti-PD-1 antibody therapy regimens are expected to be effective as a treatment strategy, the availability of a prognostic and predictive biomarker suggesting response or resistance to ovarian cancer immunotherapy based on tumor and immune expression is highly beneficial for routine clinical practice.

In this study, we report the development and results of a systematic method to leverage gene expression measured in platinum-resistant ovarian tumors by recasting individual gene transcript levels into functionally related groups that can subsequently be complementarily combined to predict a response to immune checkpoint inhibitor therapy. Despite limited sample size, we find that combinations of clear cell-specific^26^, CLOVAR immunoreactive, differentiated, mesenchymal, and proliferative^4^ gene set signatures rationally map the expression-response/resistance space for the samples available. We also identified clear cell carcinoma signature and immune-related pathways differentially expressed in CR and progressive disease (PD) subgroups. In addition to providing an interpretable and actionable result, the methodology is sufficiently straightforward that it can easily be applied to future studies with larger cohorts to improve decision-making and subsequent response rates to immune checkpoint inhibitor therapy for this challenging subclass of gynecological malignancies.

## Materials and methods

### Patient samples

Patients with platinum-resistant recurrent ovarian cancer were enrolled in a clinical trial of nivolumab, anti-PD-1 antibody, at Kyoto University from 2011 to 2015 (n = 20, UMIN000005714)^10^. The study was approved by the Kyoto University Graduate School and Faculty of Medicine, Kyoto University Hospital Ethics Committee (approval number: G531), in which donors provided written informed consent in accordance with institutional and national guidelines. Patient tumor samples were obtained by surgical dissection and stored as paraffin-fixed formalin-embedded (FFPE) tissue following recommendations of best practices for FFPE-based gene expression measurement^27^. One patient experienced thyroiditis-induced fever and tachycardia after receiving the first nivolumab dose and discontinued further treatment. This patient was excluded from the overall response analysis and data analyses, resulting in 19 patients being available for statistical investigations.

Clinical response was measured at 8 weeks following the onset of anti-PD-1 therapy (Response Evaluation Criteria in Solid Tumours (RECIST) 1.1 criteria^28^). Histological subtyping was performed. Other characteristics including but not limited to dose group, adverse events, and cancer antigen 125 levels during the course of treatment have been comprehensively presented in the literature^10^.

### Gene expression measurement

Total RNA was extracted from the FFPE tumor samples by a DNA/RNA FFPE Kit (Qiagen, Valencia, CA, USA). 90 ng of RNA were analyzed using a GeneChip Human Transcriptome Array 2.0 (microarray) in accordance with the protocol of the SensationPlus FFPE Amplification and WT Labeling Kit (Affymetrix/Thermo Fisher Scientific, Waltham, MA, USA).

### Statistical analyses

Normalization of gene expression was performed using the Single Space Transformation Robust Multi-Average (SST-RNA) method available in Expression Console v1.41. Normalized expression was quality-checked following recommendations for microarray processing^29^ and subsequently input for single-sample Gene Set Enrichment Analysis (ssGSEA)^30^. In summary, ssGSEA converts the raw expression values of a single sample into ranks based on expression value, and, for each gene set (signature) in a collection of signatures, outputs a score reflecting the relationship between the constituent genes of the signature and their ranks among all the genes in a sample. The use of multi-gene groups is advantageous in counteracting fluctuation in expression and misinterpretation of direct expression value ranks.

In the first expression analysis, we subjected patient samples to analysis by ssGSEA constrained to the application of five oncological gene signatures. Four well-studied signatures corresponding to CLOVAR immunoreactive, proliferative, mesenchymal, and differentiated gene expression subtypes in high grade serous carcinoma^4^, and a clear cell carcinoma-specific signature^26^ constituted the gene sets tested. All gene sets are in the public domain.

Values output by ssGSEA were normalized to the range 0–1 inclusive (affine transformation), per oncological signature. Significance of differential ssGSEA pathway scores amongst the CR/partial response (PR)/stable disease (SD)/PD groups was detected using a one-way analysis of variance (ANOVA) test. Rejection of the null hypothesis that all groups were equal for a given signature was determined when the probability of such (*p *value) was less than a cutoff of 0.05. As a secondary investigation, we normalized the oncological signatures by the “z-scale” transformation (subtraction from the mean and subsequent division by the standard deviation), followed by affine scaling per patient sample.

In a second gene expression analysis, the samroc method^31^ for detecting significance between pairs of groups was used to identify differential pathways. Gene sets based on the publicly available Molecular Signatures Database version 6.1 (https://software.broadinstitute.org/gsea/msigdb/), containing 21,425 pathways, were used. CR-other and PD-other analyses were initially performed, as well as a CR + PR versus SD + PD subsequent comparison necessary due to the study sample size available. The latter comparison also confirmed the sensitivity of the CR-other result as a function of the sample size. Raw probability values of null hypotheses that groups were equal (*p *values) were compensated for multiple hypothesis testing (of the 21,425 pathways) by using the “p. adjust” function built into the R statistical environment (http://www.r-project.org), resulting in false discovery rate (FDR) q-values. A threshold of q < 0.05 was used to reject the null hypothesis.

### Weights in fitting signature scores versus clinical response

Weights for a linear combination of the five ovarian cancer-specific signatures were obtained by solving a least-squares fitting equation a * X = b available from the “linalg.lstsq” solver built into the NumPy matrix processing library (www.scipy.org), an extension of the Python programming language. The matrix **X** contained the raw 0–1 ssGSEA scores as described above and therefore was of shape 19 × 5. The vector **b** (of length 19) was set to follow the RECIST 1.1 criteria for changes in tumor volume, and therefore, quantities in **b** corresponding to tumor growth evaluation criteria were PD = 0.2, SD = 0, PR =  − 0.3, and CR =  − 1.0.

### Ethics approval and consent to participate

This study was approved by the Kyoto University Graduate School and Faculty of Medicine, Kyoto University Hospital Ethics Committee (Approval Number: G531), in which donors provided written informed consent in accordance with institutional and national guidelines.

## Results

### Correlation between gene signature groups and clinical response

Nineteen patients, histologically comprising 14 with high-grade serous carcinomas (73%), three with endometrioid carcinomas (16%), and two with clear cell carcinomas (11%), were analyzed; these ratios were concordant with prior publication^1^. There were two patients with CR, one with PR, six with SD, and 10 with PD; the response rate was consistent with that of an independent study^16^. One patient demonstrated a complete elimination of the primary target lesion but experienced a para-aortic metastasis. Therefore, this patient was given a special designation of “SD (CR)” while being allocated in the PR group for signature-response analyses. The pair of complete responders had CA125 levels that exponentially decayed over the time course of treatment.

**Figure 1:**
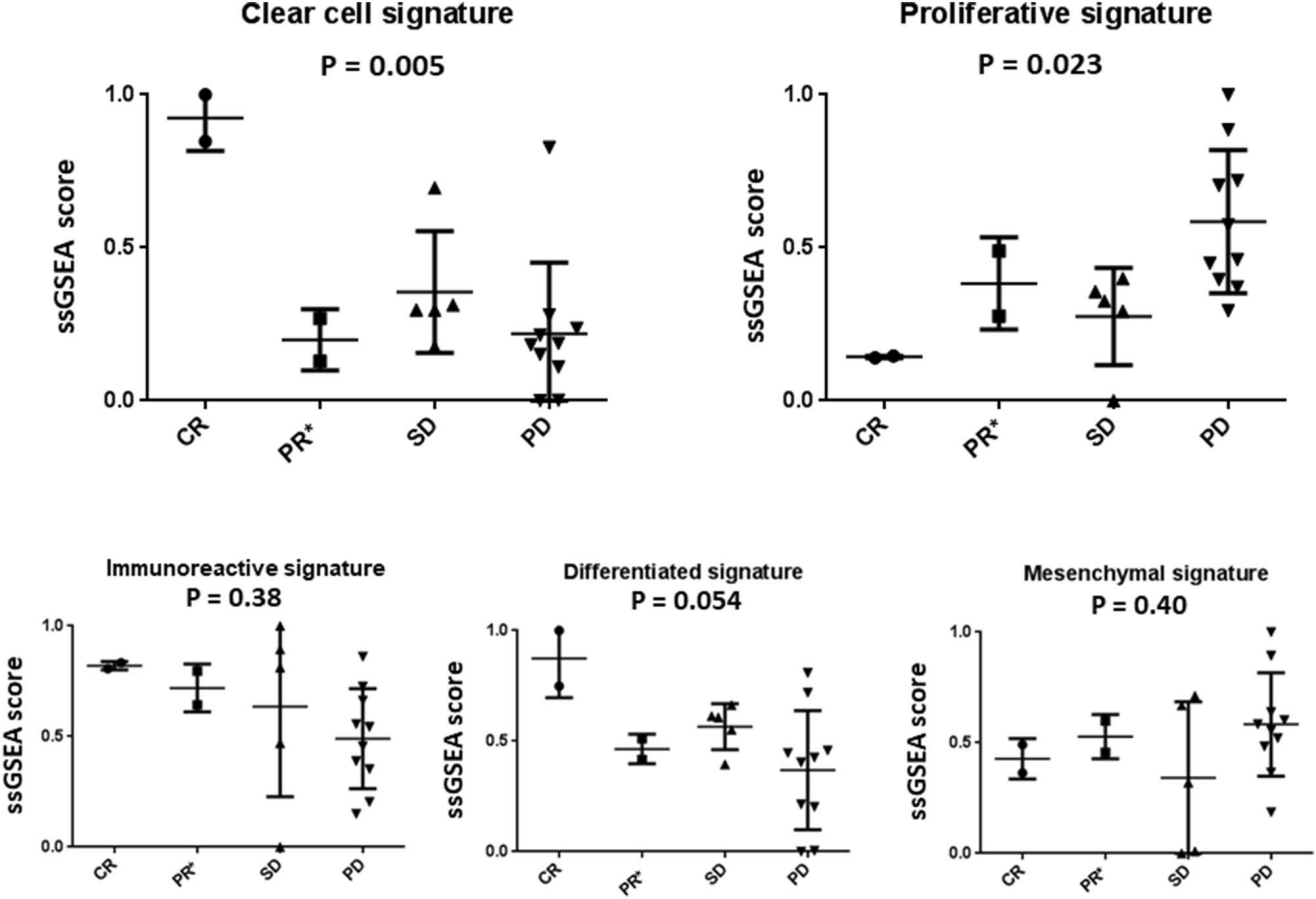
Significance testing of ovarian signature scores stratified by clinical response. ssGSEA was applied with gene signature sets tailored to ovarian cancer, resulting in per-signature scores normalized to the range 0–1. The clear cell signature is significant in complete responders and the proliferative signature was significantly different in patients who exhibited progressive disease. The PR* group comprises one patient classified as PR by RECIST protocols and one exceptional patient (see “Methods”). Image was created using Prism version 8.3.0 (https://www.graphpad.com/).

ANOVA analysis between tumors of the response groups revealed that the pair of patients with CR had significantly increased clear cell gene signature scores (Fig. 1 upper left, *p* = 0.005). Aside from one PD patient whose expression yielded a high clear cell score, scores of the PD patients were systemically reduced. Conversely, tumors from the PD patients had significantly higher CLOVAR proliferative gene signature scores compared to those from the other groups (Fig. 1 upper right, *p* = 0.026). There was no overlap in the proliferative signature score values or confidence intervals between the CR and PD groups. Considering these results, the clear cell and proliferative gene signatures are suggestive of positive or negative response to anti-PD-1 antibody treatment in ovarian cancer.

Although not statistically significant at a cutoff value of *p* = 0.05, clear trends were observed in the scores of the other signatures tested. These included the increased immunoreactive signature scores in the patients with CR and PR (Fig. 1, bottom left), the higher differentiation signature scores in the patients with CR (Fig. 1, bottom center, *p* = 0.054), and the increased mesenchymal signature scores in the patients with PD (Fig. 1, bottom right).

For reference, we also performed signature significance testing by stratifying samples into their clear cell, serous, and endometrial histopathological subtypes. A figure analogous to Fig. 1 is provided as Supplementary Fig. S1. Besides the anticipated result that the clear cell samples had higher clear cell signature scores than the other samples, there were no significant correlations. One patient with a serous carcinoma also exhibited hallmarks of the clear cell subtype when a sample of the tumor was viewed under the microscope; this patient had an increased clear cell signature score (Supplementary Fig. S1).

### Mutual complementarity effects of gene signature groups

**Figure 2:**
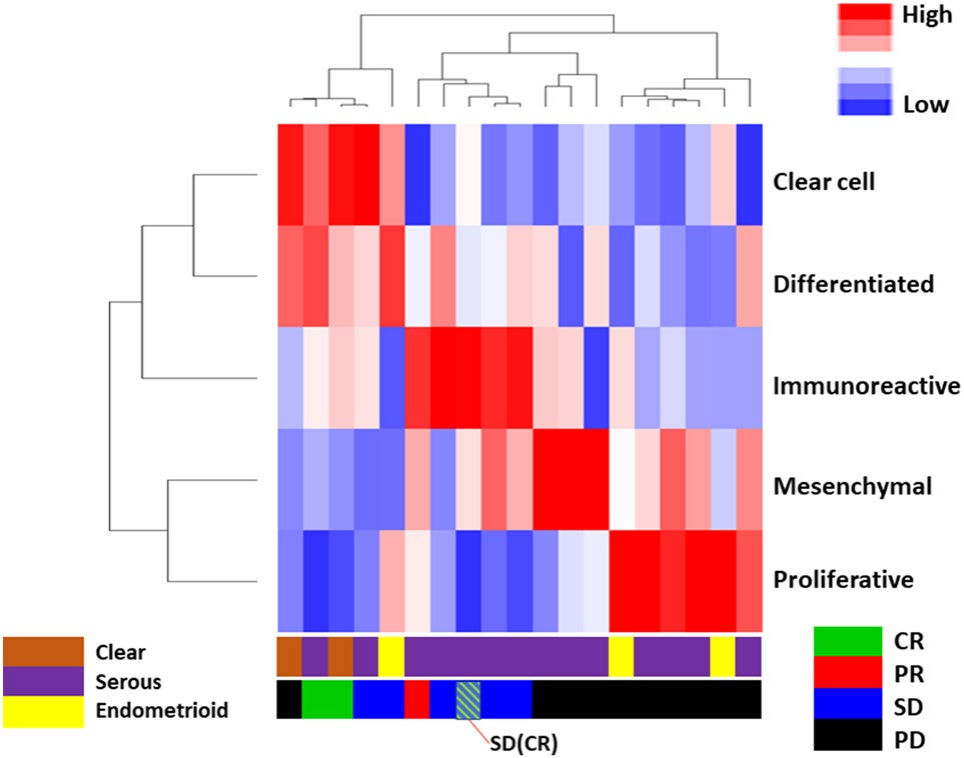
Complementarity of gene signatures in predicting clinical response. Gene signature groups were biclustered, and patient clinical responses were annotated. An additional annotation track of histopathological subtype is provided. Score values were normalized by centering on the mean for each signature, and average linkage was used to create the dendrogram. Combinations of signature groups reinforce prediction of clinical response to therapy. Image was created using Python 3.0. (https://www.python.org/download/releases/3.0/).

Unsupervised hierarchical clustering of patients and signatures was performed (Fig. 2). After additional annotation with clinical response, a reasonable trend between the signature groups and their response was obtained. Although the immunoreactive, differentiated, and mesenchymal gene signatures did not yield probability values that passed the *p* = 0.05 threshold to be considered significant (Fig. 1), Fig. 2 suggests that the combination of both significant and statistically nonsignificant gene signature scores provides complementary points of evidence and improved decision-making support over the evidence suggested by any individual signature score. For example, although the patients with PD dominantly had increased scores in the proliferative gene signature, consideration of the proliferative and mesenchymal signatures in parallel provided more complete coverage of the PD patients than either signature alone. Consideration of the combined clear cell, differentiated, and immunoreactive signatures better suggests a response (non-PD) than any isolated signature value. Similar heatmaps with patients grouped by clinical response or by an additional per-patient normalization prior to clustering are available in Supplementary Fig. S2 and Supplementary Fig. S3, respectively.

Based on the trends of Figs. 1, 2, Supplementary Fig. S2, and Supplementary Fig. S3, we attempted to quantify the weights for each signature score that could subsequently be combined to forecast response to nivolumab. By solving a least-squares fit of the score-response data, we derived the following response predictor with a functional form:

$$\begin{aligned} RESP \, & = - 0.439 \, \times \, CLE \, + \, 0.013 \, \times \, DIFF \\ & \quad - 0.364 \, \times \, IMR \, + \, 0.308 \, \times \, MES \, + \, 0.350 \, \times \, PRO \\ \end{aligned}$$

where a negative response score indicates a positive response to treatment (reduction in tumor volume) and a positive score indicates a lack of response. Further analyses confirmed that the weights were invariant to any uniform scaling of the response group categories. This formula could be applied as-is in a clinical setting when tumor bulk RNA expression is measured and ssGSEA signature values are computed from the result. A new fitting of weight constants (**a**) can be updated upon acquisition of new data simply by extending the patient-signature matrix (**X**) and the vector of patient responses (**b**).

**Figure 3:**
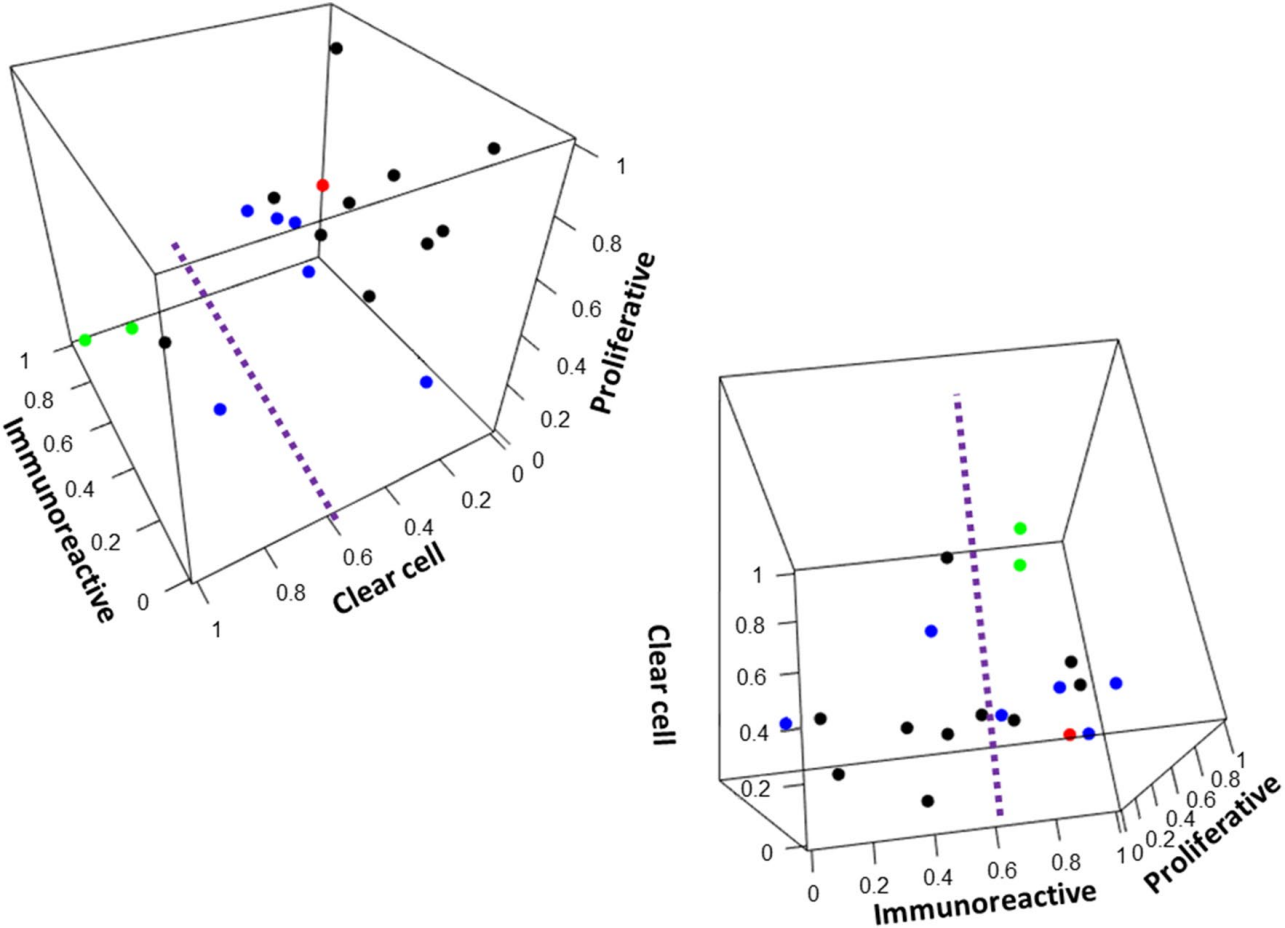
Three-dimensional signature score projection. Expansion from two-dimensional to three-dimensional projection improves response demarcation. A 360-degree viewpoint animation of the project is available as supplementary data. Points are colored using the same annotation as Fig. 1. Figure 3 image was created using R statistical environment version 3.6.0 (http://www.r-project.org).

A visual interpretation of a patient’s ssGSEA scores is helpful to both the physician and the patient. Given the signs and magnitudes of the weights computed in the formula above, analyses were subsequently performed to project a combination of the three gene signature scores as a point in a three-dimensional coordinate space, with coloring of points by clinical response. As demonstrated in Fig. 3, a projection using the combination of immunoreactive, proliferative, and clear cell signatures provides a quick visual guide to the prediction of antitumor response via spatial clustering. Supplementary Fig. S4 is an animation demonstrating a 360-degree view of this three-dimensional projection.

For further analysis, ssGSEA values of individual signatures (Fig. 1) were evaluated by histogram, and their pairwise correlations were computed by Pearson’s product moment correlation (Supplementary Fig. S5). We observed from the individual signature score distributions that the clear cell gene signature is distinctively bimodal, whereas the CLOVAR subtype signatures follow more of a Gaussian or skewed distribution. With respect to the pairwise signature correlation, a negative correlation (r =  − 0.69) was observed between the proliferative and clear cell gene signatures, which is an anticipated finding but critically serves as a necessary negative control for the investigation. A positive correlation between the differentiated and clear cell signatures (r = 0.65) supports the view that clear cell carcinoma is a well-differentiated subtype, as previously reported^32^. When restricted to genes reported as upregulated in the literature, the differential-clear cell signature correlation increases (r = 0.75).

### Differential pathways in response groups

A secondary ssGSEA analysis was performed to search for additional gene signatures and pathways that correlated with patient responses. As the CR and PD patients were significantly different in ANOVA analyses based on the CLOVAR and clear cell signatures (Fig. 1), CR-vs-other and PD-vs-other samroc analyses were performed for 21,425 pathways (see “Materials and Methods” section).

Using the collection of genes that had higher average expression in the CR patients than in the other response groups, samroc analysis corroborated the effectiveness of the clear cell-specific signature (Supplementary Table S1). In the downregulated genes of the CR group, pathways associated with positive regulation of stem cell differentiation and epithelial-to-mesenchymal transition were detected as significant (Supplementary Table S2), thus inferring the suppression of these well-known tumor growth processes.

From the tissues of the PD patients, samroc analysis of upregulated genes identified not only the serous-specific proliferative gene signature but also gene sets characterizing pathways for positive regulation of extracellular matrix organization and mesenchymal cell proliferation (Supplementary Table S3). The PD group also demonstrated downregulation of pro-inflammatory cytokines, including tumor necrosis factor binding, regulation of interleukin synthesis, cytokine secretion and inflammatory response, and B-cell mediated immunity (Supplementary Table S4).

We repeated the analyses by combining the CR group and the partial responder and querying how the samroc detection of pathways would be affected. Although some new pathways emerged as significant only in the CR + PR upregulated gene group, there was a reasonable overlap within the original CR-only analysis, including the clear cell signature (Supplementary Fig. S6 and Supplementary Table S5). Analysis of the downregulated genes in the CR + PR group returned pathways having more overlap with the CR-only group than the number of new pathways unique to only the CR + PR group. Those commonly downregulated pathways once again included regulation of stem cell differentiation, mesenchymal-to-epithelial transition, and regulation of cell–cell adhesion mediated by cadherin (Supplementary Fig. S7 and Supplementary Table S6).

## Discussion

Ovarian cancer has proven to be a challenging cancer type to treat, as it has multiple subtypes that confer differing responses to therapy. The platinum-taxane regimen was conceived with the hypothesis that it could cover the variety of subtypes, although resistance to the regimen is common. We have previously shown that the mesenchymal subtype was sensitive to taxane but not to platinum, whereas the proliferative subtype was sensitive to platinum^5^. The introduction of targeted agents such as bevacizumab has shown improved response in proliferative subtype patients^33^ and platinum-resistant mesenchymal subtype patients. The mechanistic role of immune checkpoint inhibitors in the subtype-therapy-response dynamic and the effectively selection of patients has been an open question.

In this study, we thus aimed to establish a systematic and reusable method to capture a systems-level biological insight from a limited number of ovarian tumor transcriptomes. Individual gene sets demonstrated informative trends, and could be combined into an interpretable linear combination to contribute to the holistic subtype-therapy-response dynamic in the context of nivolumab. The obtained linear combination readily and significantly explained the outcomes of the PD group in the proliferative and mesenchymal subtypes, which have poor prognoses. These two subtypes are currently being addressed with the use of bevacizumab^33^. Additionally, the formula indicated that response to nivolumab could be expected through increased immunoreactivity and clear cell signature scores. This latter fact is particularly interesting, as it covers the subtypes in which bevacizumab is not efficacious.

Due to a number of factors beyond our control, the study had a limitation in that it had a small sample size, and our findings must be contextualized as a prospect. Nonetheless, in combination with recent reports of ovarian cancer therapy response studies, immune checkpoint inhibitor therapy and the subtype-treatment-response dynamic can be enriched by our results.

Recently, Moore et al. presented the first results of the phase III IMagyn050/GOG3015/ENGOT-OV39 trial. In this trial, the addition of atezolizumab to bevacizumab and platinum-taxane chemotherapy failed to significantly improve progression-free survival (PFS) in patients with newly diagnosed advanced ovarian cancer. However, results from exploratory PFS analyses demonstrated a trend favoring atezolizumab in a subgroup of patients with 5% or more PD-L1-expressive immune cells. Here, the median PFS with the atezolizumab regimen was not obtained, as compared to the median of 20.2 months in the bevacizumab/chemotherapy arm (hazard ratio [HR] 0.64; 95% confidence interval [CI] 0.43–0.96; significant). Furthermore, in histological subgroup analyses, patients with clear cell carcinoma showed favorable PFS (HR 0.64; 95% CI 0.33–1.24; not significant) in the atezolizumab arm (n = 29) compared to the placebo arm (n = 22), which could warrant further evaluation due to small sample size^34^. These findings of clinical benefit via atezolizumab in the immune cell PD-L1-high subgroup and in the patients with clear cell carcinoma are consistent with our results that response to nivolumab could be expected through elevated immunoreactivity and clear cell phenotype.

Additional studies on the general strategy of treatment by combinations of small molecule and immune checkpoint inhibitor antibodies are underway. For example, an investigation in recurrent platinum-resistant ovarian cancer therapy by combining the recent PARP inhibitor niraparib with pembrolizumab has recently been reported^35^. A major effort for advanced ovarian cancer tumor therapy using targeted small molecule inhibitors with a candidate anti-PD-1 antibody is also ongoing (NCT03737643, ClinicalTrials.gov). The study evaluates the use of durvalumab treatment in combination with chemotherapy and bevacizumab, followed by maintenance durvalumab, bevacizumab, and olaparib treatment^36^. When conducted with appropriate biobanking techniques, these and similar studies could immediately apply the methodologies we have proposed in this report and eventually produce novel biomarkers for improved clinical decision-making, combining with DNA sequencing analyses such as tumor mutation burden or microsatellite instability analysis. These advances are highly needed in the study of ovarian cancer to populate the data space of the subtype-therapy-response dynamic.

In conclusion, the paired gene signature and pathway analyses have provided starting ground for developing reliable predictors of clinical response to immune checkpoint inhibitor therapy in ovarian cancer. The collection of signature scores holistically correlated with clinical response to anti-PD-1 antibody therapy, and the mathematical model developed could be implemented for immediate clinical application. Aggregation of cases will contribute to the fine-tuning of the subtype-therapy-response dynamic established in this study.

## Supplementary Information

Supplementary Information 1.

Supplementary Information 2.

Supplementary Information 3.

Supplementary Information 4.

Supplementary Information 5.

Supplementary Information 6.

Supplementary Information 7.

Supplementary Information 8.

Supplementary Information 9.

Supplementary Information 10.

Supplementary Information 11.

Supplementary Information 12.

Supplementary Information 13.

Supplementary Information 14.

## Data Availability

The datasets generated and analyzed during the current study are available from the corresponding author upon reasonable request.

## References

[1] Prat J (2012). New insights into ovarian cancer pathology. Ann. Oncol..

[2] Sugiyama T (2000). Clinical characteristics of clear cell carcinoma of the ovary: a distinct histologic type with poor prognosis and resistance to platinum-based chemotherapy. Cancer.

[3] Sung PL, Chang YH, Chao KC, Chuang CM (2014). Global distribution pattern of histological subtypes of epithelial ovarian cancer: a database analysis and systematic review. Gynecol. Oncol..

[4] Verhaak RG (2013). Prognostically relevant gene signatures of high-grade serous ovarian carcinoma. J. Clin. Investig..

[5] Murakami R (2016). Prediction of taxane and platinum sensitivity in ovarian cancer based on gene expression profiles. Gynecol. Oncol..

[6] Murakami R (2019). The mesenchymal transition subtype more responsive to dose dense taxane chemotherapy combined with carboplatin than to conventional taxane and carboplatin chemotherapy in high grade serous ovarian carcinoma: a survey of Japanese Gynecologic Oncology Group study (JGOG3016A1). Gynecol. Oncol..

[7] Murakami R (2016). Establishment of a novel histopathological classification of high-grade serous ovarian carcinoma correlated with prognostically distinct gene expression subtypes. Am. J. Pathol..

[8] Galon J, Bruni D (2019). Approaches to treat immune hot, altered and cold tumours with combination immunotherapies. Nat. Rev. Drug Discov..

[9] Liu JF (2019). Assessment of combined nivolumab and bevacizumab in relapsed ovarian cancer: A phase 2 clinical trial. JAMA Oncol..

[10] Hamanishi J (2015). Safety and antitumor activity of anti-PD-1 antibody, nivolumab, in patients with platinum-resistant ovarian cancer. J. Clin. Oncol..

[11] Mahoney KM, Freeman GJ, McDermott DF (2015). The next immune-checkpoint inhibitors: PD-1/PD-L1 blockade in melanoma. Clin. Ther..

[12] Snyder A (2014). Genetic basis for clinical response to CTLA-4 blockade in melanoma. N. Engl. J. Med..

[13] Guibert N, Mazieres J (2015). Nivolumab for treating non-small cell lung cancer. Expert Opin. Biol. Ther..

[14] Patel SP, Kurzrock R (2015). PD-L1 expression as a predictive biomarker in cancer immunotherapy. Mol. Cancer Ther..

[15] Callahan MK, Postow MA, Wolchok JD (2016). Targeting T cell co-receptors for cancer therapy. Immunity.

[16] Varga A (2019). Pembrolizumab in patients with programmed death ligand 1-positive advanced ovarian cancer: analysis of KEYNOTE-028. Gynecol. Oncol..

[17] Hamanishi J (2016). PD-1/PD-L1 blockade in cancer treatment: perspectives and issues. Int. J. Clin. Oncol..

[18] Motzer RJ (2015). Nivolumab versus everolimus in advanced renal-cell carcinoma. N. Engl. J. Med..

[19] Borghaei H (2015). Nivolumab versus docetaxel in advanced nonsquamous non-small-cell lung cancer. N. Engl. J. Med..

[20] Ansell SM (2015). PD-1 blockade with nivolumab in relapsed or refractory Hodgkin's lymphoma. N. Engl. J. Med..

[21] Iwai Y, Hamanishi J, Chamoto K, Honjo T (2017). Cancer immunotherapies targeting the PD-1 signaling pathway. J. Biomed. Sci..

[22] Cogdill AP, Andrews MC, Wargo JA (2017). Hallmarks of response to immune checkpoint blockade. Br. J. Cancer.

[23] Doo DW, Norian LA, Arend RC (2019). Checkpoint inhibitors in ovarian cancer: a review of preclinical data. Gynecol. Oncol. Rep..

[24] Cristescu, R. *et al.* Pan-tumor genomic biomarkers for PD-1 checkpoint blockade-based immunotherapy. *Science (New York, N.Y.)* **362**. 10.1126/science.aar3593 (2018). 10.1126/science.aar3593 PMC6718162 30309915

[25] Ott PA (2019). T-cell-inflamed gene-expression profile, programmed death ligand 1 expression, and tumor mutational burden predict efficacy in patients treated with pembrolizumab across 20 cancers: KEYNOTE-028. J. Clin. Oncol..

[26] Yamaguchi K (2010). Identification of an ovarian clear cell carcinoma gene signature that reflects inherent disease biology and the carcinogenic processes. Oncogene.

[27] Belder N (2016). From RNA isolation to microarray analysis: comparison of methods in FFPE tissues. Pathol. Res. Pract..

[28] Eisenhauer, E. A. *et al.* New response evaluation criteria in solid tumours: revised RECIST guideline (version 1.1). *Eur. J. Cancer (Oxford, England : 1990)* **45**, 228–247.10.1016/j.ejca.2008.10.026 (2009). 10.1016/j.ejca.2008.10.026 19097774

[29] Yu J (2015). Multi-platform assessment of transcriptional profiling technologies utilizing a precise probe mapping methodology. BMC Genomics.

[30] Barbie DA (2009). Systematic RNA interference reveals that oncogenic KRAS-driven cancers require TBK1. Nature.

[31] Broberg P (2003). Statistical methods for ranking differentially expressed genes. Genome Biol..

[32] Winterhoff B (2016). Molecular classification of high grade endometrioid and clear cell ovarian cancer using TCGA gene expression signatures. Gynecol. Oncol..

[33] Kommoss S (2017). Bevacizumab may differentially improve ovarian cancer outcome in patients with proliferative and mesenchymal molecular subtypes. Clin. Cancer Res..

[34] Moore, K. N. *et al.* Atezolizumab, bevacizumab, and chemotherapy for newly diagnosed stage III or IV ovarian cancer: placebo-controlled randomized phase III trial (IMagyn050/GOG 3015/ENGOT-OV39). *J. Clin. Oncol.*10.1200/jco.21.00306 (2021). 10.1200/JCO.21.00306 PMC8189598 33891472

[35] Konstantinopoulos PA (2019). Single-arm phases 1 and 2 trial of niraparib in combination with pembrolizumab in patients with recurrent platinum-resistant ovarian carcinoma. JAMA Oncol..

[36] Harter, P. *et al.* DUO-O: a randomized phase III trial of durvalumab (durva) in combination with chemotherapy and bevacizumab (bev), followed by maintenance durva, bev and olaparib (olap), in newly diagnosed advanced ovarian cancer patients. *J. Clin. Oncol.* **37**, TPS5598–TPS5598. 10.1200/JCO.2019.37.15_suppl.TPS5598 (2019).

