# Supplementary material for: Combination of gene set signatures correlates with response to nivolumab in platinum-resistant ovarian cancer
Source: Sci Rep. 2021 Jun 1;11:11427. doi: 10.1038/s41598-021-91012-w (PMC8169687; doi:10.1038/s41598-021-91012-w)
Supplement: Supplementary file 1 — Supplementary Information 1. [file 41598_2021_91012_MOESM1_ESM.pptx]

## Slide 1
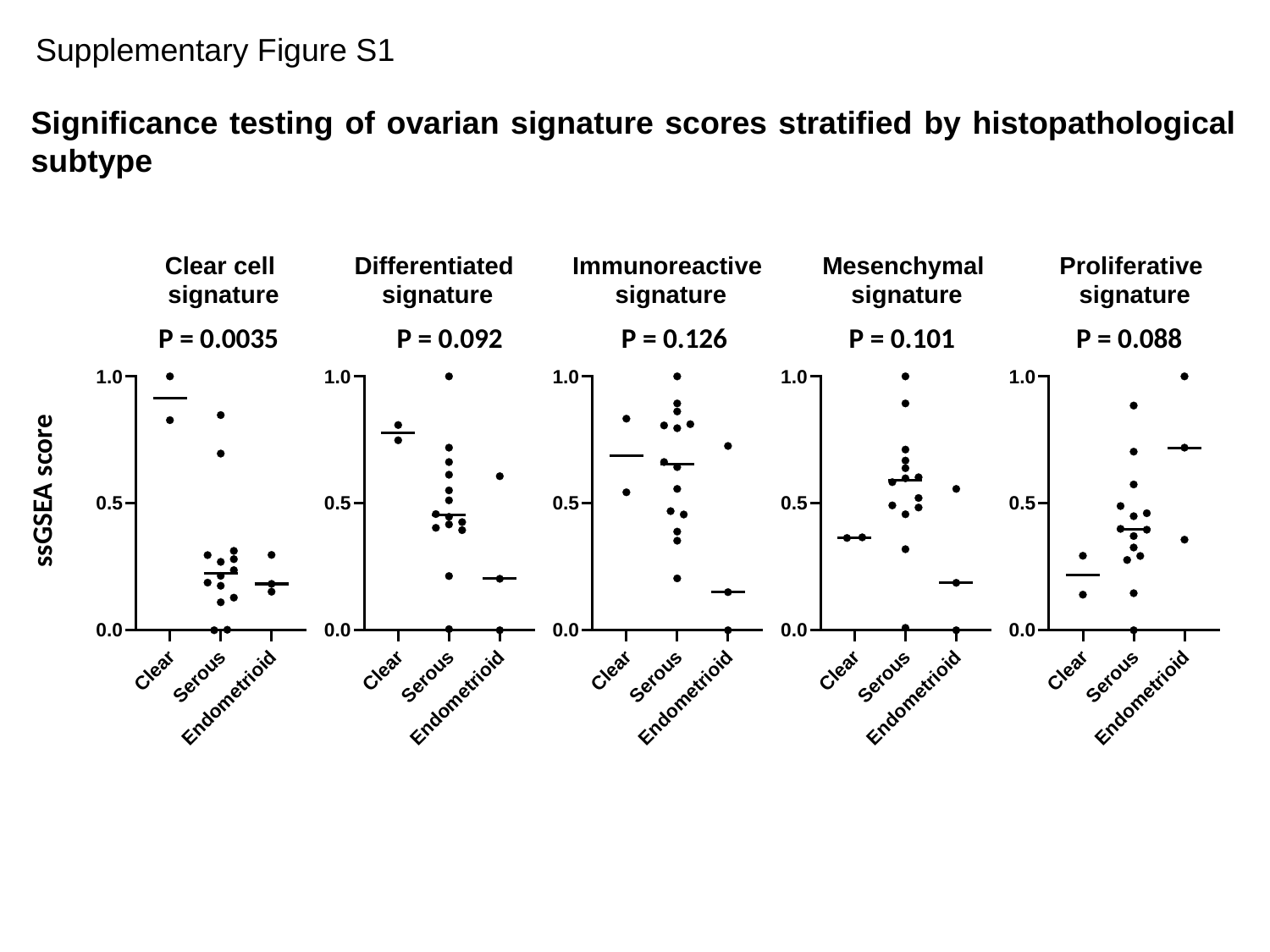

Supplementary Figure S1
Significance testing of ovarian signature scores stratified by histopathological subtype
Clear cell
 signature
Proliferative
 signature
Differentiated
 signature
Immunoreactive
 signature
Mesenchymal
 signature
P = 0.0035
P = 0.092
P = 0.126
P = 0.101
P = 0.088
ssGSEA score
