# Supplementary material for: Combination of gene set signatures correlates with response to nivolumab in platinum-resistant ovarian cancer
Source: Sci Rep. 2021 Jun 1;11:11427. doi: 10.1038/s41598-021-91012-w (PMC8169687; doi:10.1038/s41598-021-91012-w)
Supplement: Supplementary file 2 — Supplementary Information 2. [file 41598_2021_91012_MOESM2_ESM.pptx]

## Slide 1
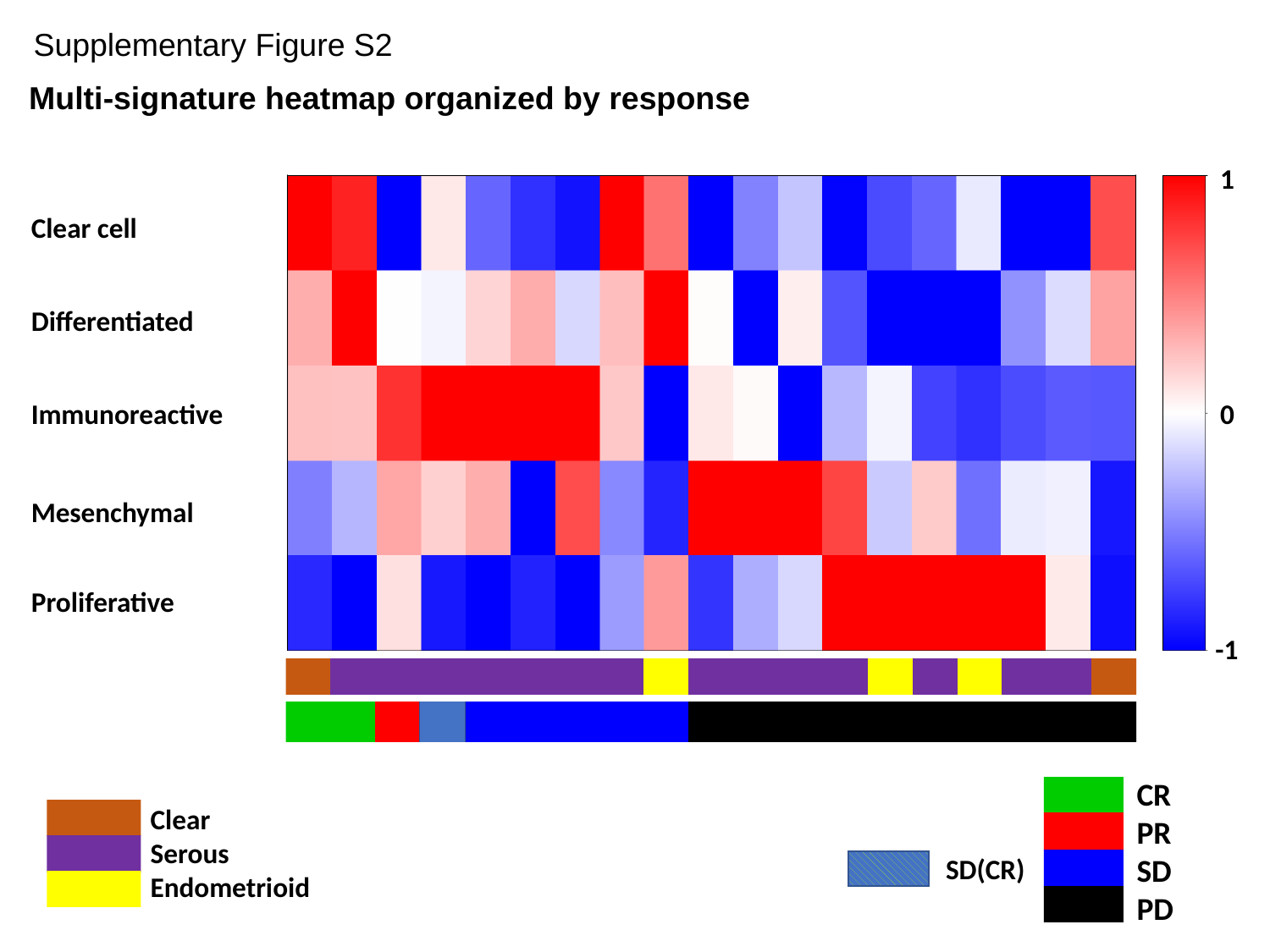

Supplementary Figure S2
Multi-signature heatmap organized by response
1
Clear cell
Differentiated
0
Immunoreactive
Mesenchymal
Proliferative
-1
CR
PR
SD
PD
Clear
Serous
Endometrioid
SD(CR)
