# Supplementary material for: Combination of gene set signatures correlates with response to nivolumab in platinum-resistant ovarian cancer
Source: Sci Rep. 2021 Jun 1;11:11427. doi: 10.1038/s41598-021-91012-w (PMC8169687; doi:10.1038/s41598-021-91012-w)
Supplement: Supplementary file 3 — Supplementary Information 3. [file 41598_2021_91012_MOESM3_ESM.pptx]

## Slide 1
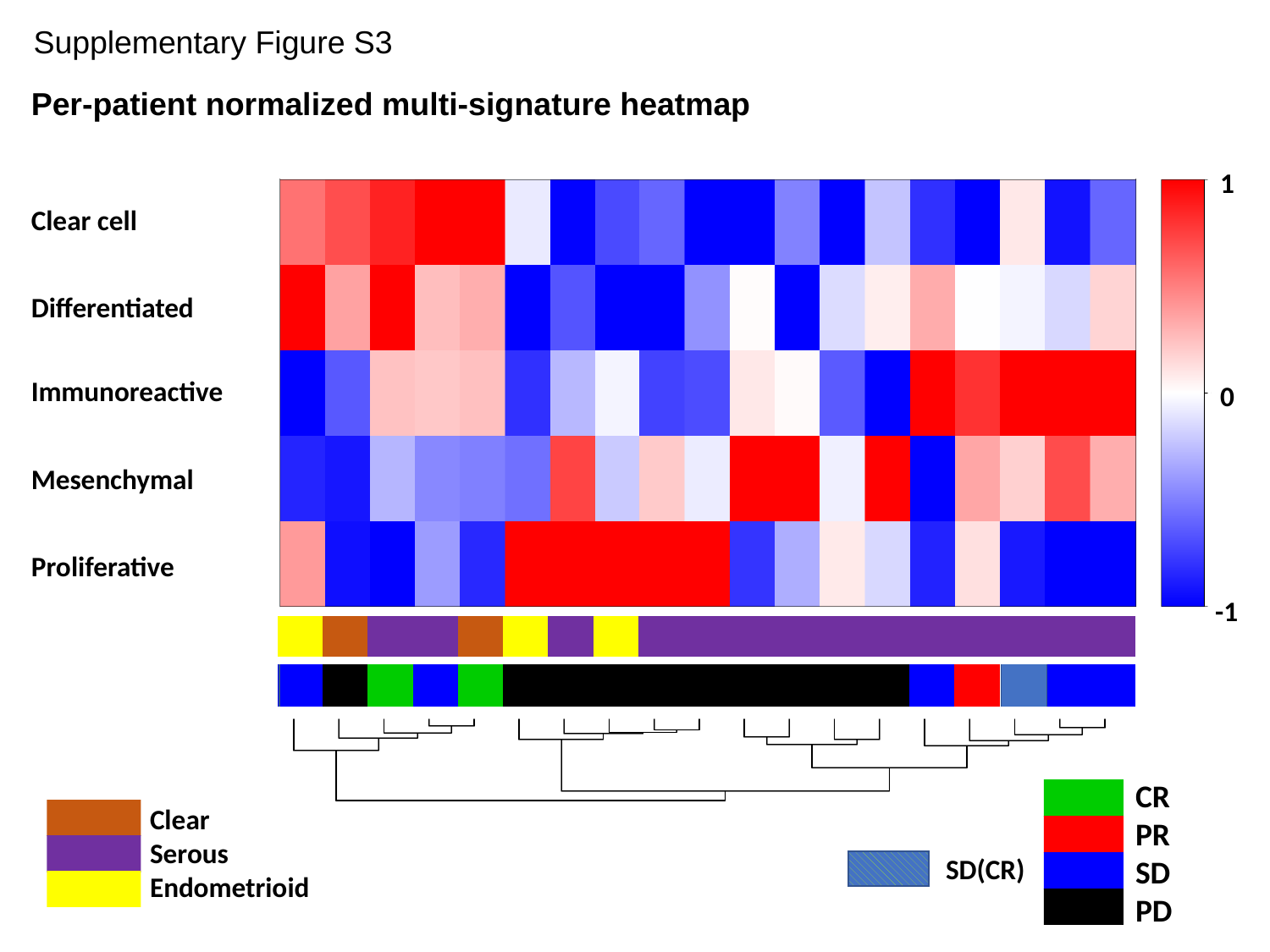

Supplementary Figure S3
Per-patient normalized multi-signature heatmap
1
Clear cell
Differentiated
Immunoreactive
0
Mesenchymal
Proliferative
-1
CR
PR
SD
PD
Clear
Serous
Endometrioid
SD(CR)
