# Supplementary figures and images for: Combination of gene set signatures correlates with response to nivolumab in platinum-resistant ovarian cancer
Source: Sci Rep. 2021 Jun 1;11:11427. doi: 10.1038/s41598-021-91012-w (PMC8169687; doi:10.1038/s41598-021-91012-w)

## Slide 1
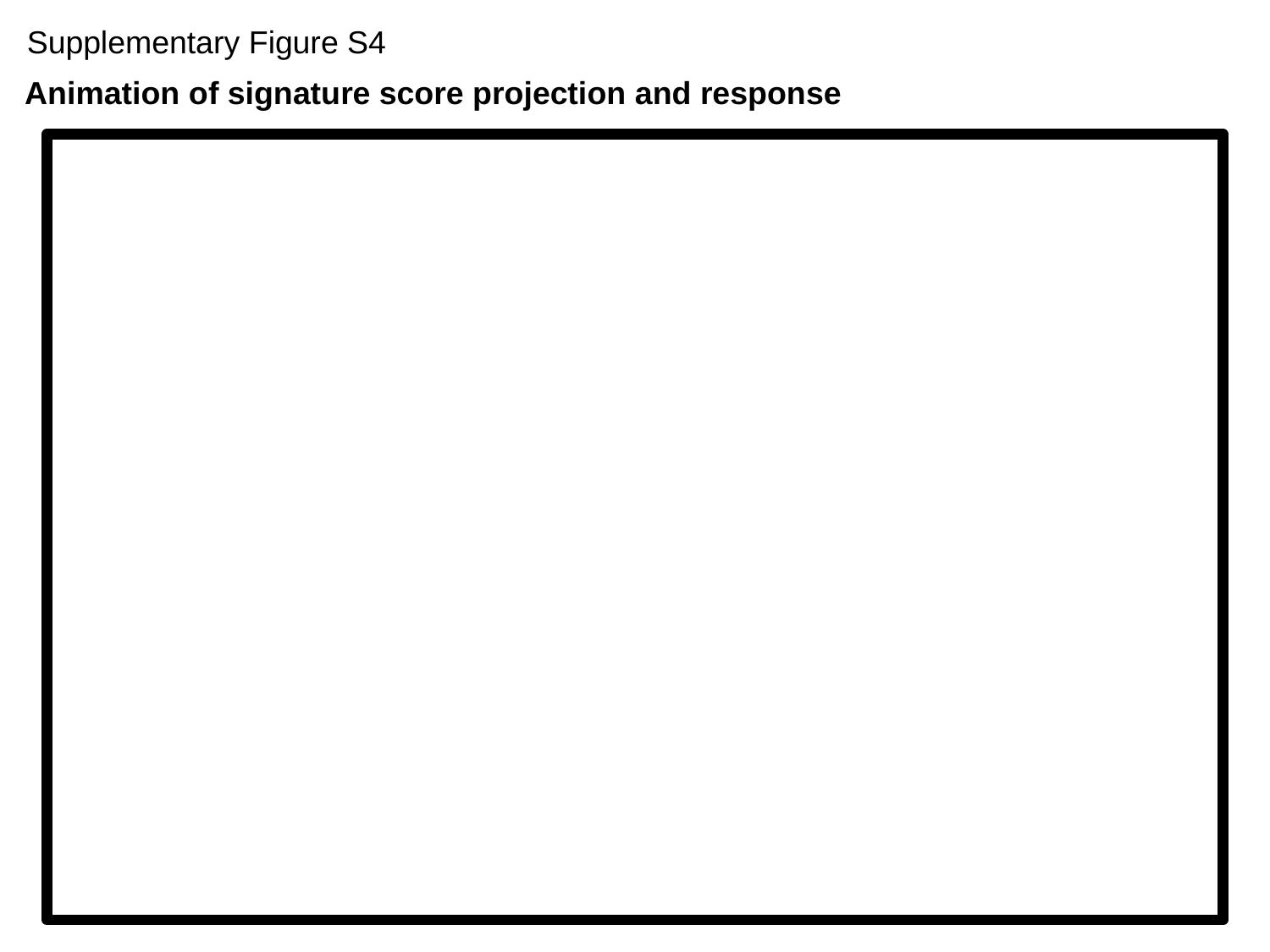

Supplementary Figure S4
Animation of signature score projection and response

Supplement: Supplementary file 4 — Supplementary Information 4. [file 41598_2021_91012_MOESM4_ESM.pptx]
