# Supplementary material for: Combination of gene set signatures correlates with response to nivolumab in platinum-resistant ovarian cancer
Source: Sci Rep. 2021 Jun 1;11:11427. doi: 10.1038/s41598-021-91012-w (PMC8169687; doi:10.1038/s41598-021-91012-w)
Supplement: Supplementary file 5 — Supplementary Information 5. [file 41598_2021_91012_MOESM5_ESM.pptx]

## Slide 1
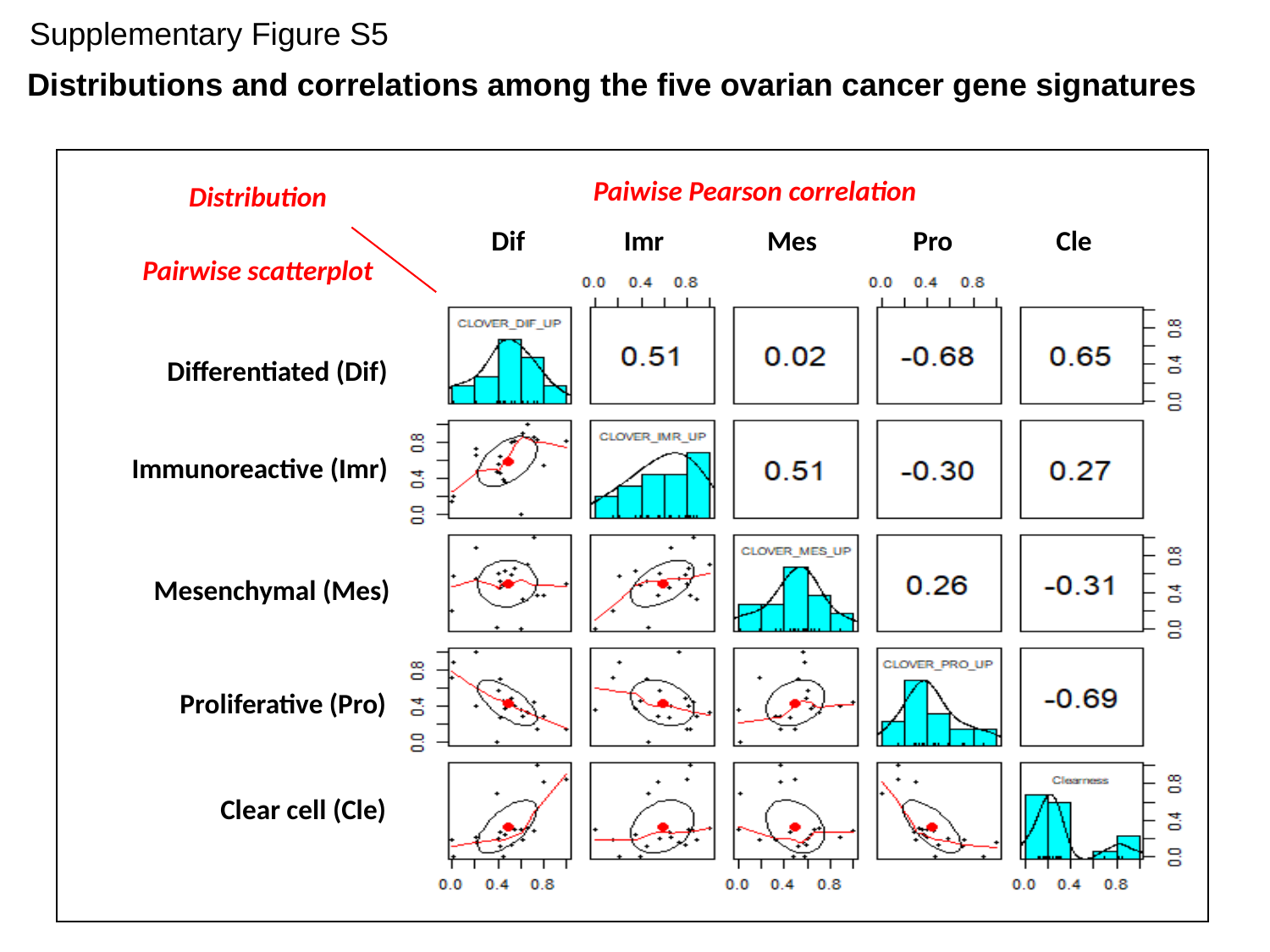

Supplementary Figure S5
Distributions and correlations among the five ovarian cancer gene signatures
Paiwise Pearson correlation
Distribution
Dif
Imr
Mes
Pro
Cle
Pairwise scatterplot
Differentiated (Dif)
Immunoreactive (Imr)
Mesenchymal (Mes)
Proliferative (Pro)
Clear cell (Cle)
