# Supplementary material for: Combination of gene set signatures correlates with response to nivolumab in platinum-resistant ovarian cancer
Source: Sci Rep. 2021 Jun 1;11:11427. doi: 10.1038/s41598-021-91012-w (PMC8169687; doi:10.1038/s41598-021-91012-w)
Supplement: Supplementary file 6 — Supplementary Information 6. [file 41598_2021_91012_MOESM6_ESM.pptx]

## Slide 1
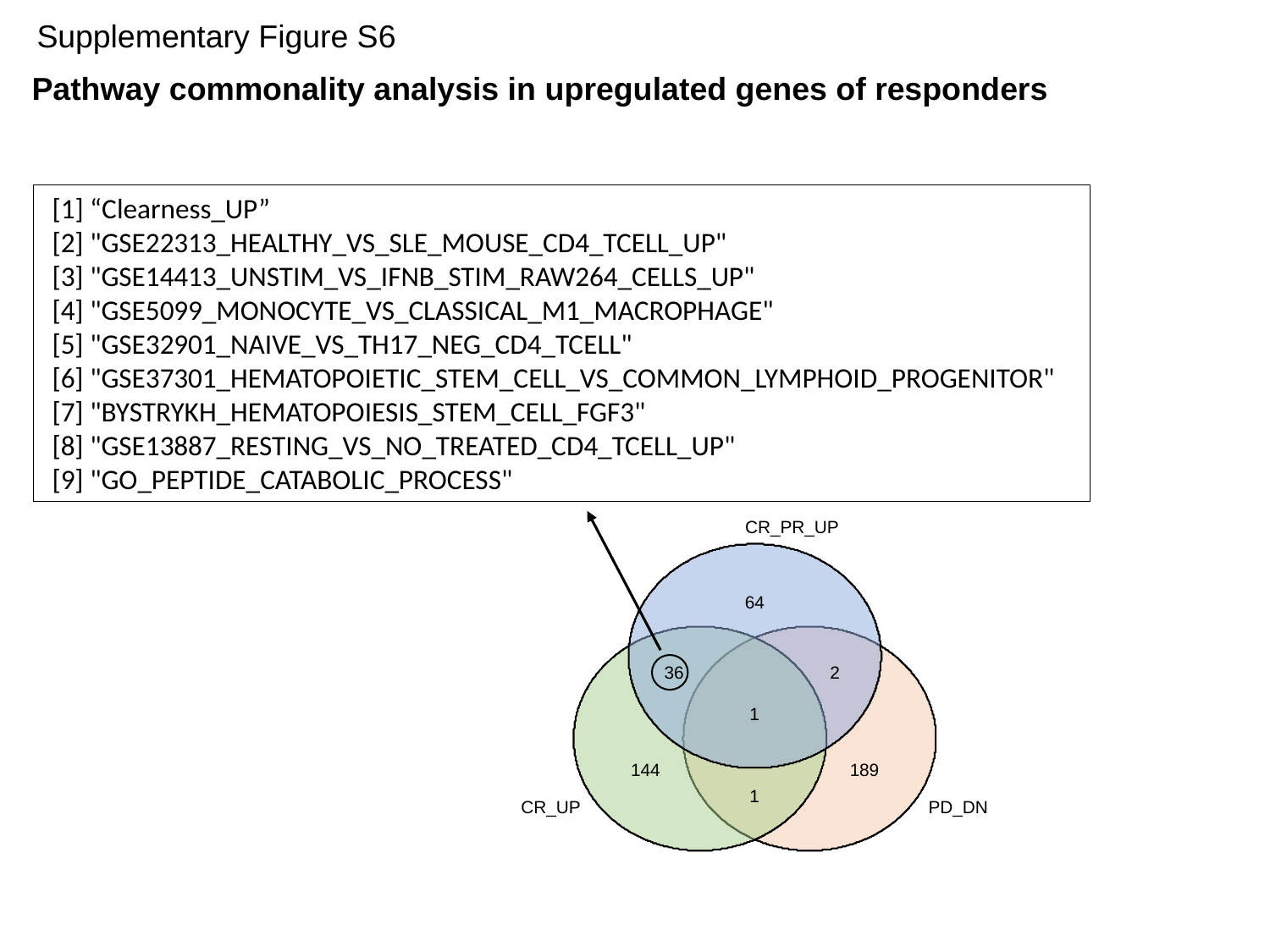

Supplementary Figure S6
Pathway commonality analysis in upregulated genes of responders
 [1] “Clearness_UP”
 [2] "GSE22313_HEALTHY_VS_SLE_MOUSE_CD4_TCELL_UP"
 [3] "GSE14413_UNSTIM_VS_IFNB_STIM_RAW264_CELLS_UP"
 [4] "GSE5099_MONOCYTE_VS_CLASSICAL_M1_MACROPHAGE"
 [5] "GSE32901_NAIVE_VS_TH17_NEG_CD4_TCELL"
 [6] "GSE37301_HEMATOPOIETIC_STEM_CELL_VS_COMMON_LYMPHOID_PROGENITOR"
 [7] "BYSTRYKH_HEMATOPOIESIS_STEM_CELL_FGF3"
 [8] "GSE13887_RESTING_VS_NO_TREATED_CD4_TCELL_UP"
 [9] "GO_PEPTIDE_CATABOLIC_PROCESS"
CR_PR_UP
64
36
2
1
144
189
1
CR_UP
PD_DN
