# Supplementary material for: Combination of gene set signatures correlates with response to nivolumab in platinum-resistant ovarian cancer
Source: Sci Rep. 2021 Jun 1;11:11427. doi: 10.1038/s41598-021-91012-w (PMC8169687; doi:10.1038/s41598-021-91012-w)
Supplement: Supplementary file 7 — Supplementary Information 7. [file 41598_2021_91012_MOESM7_ESM.pptx]

## Slide 1
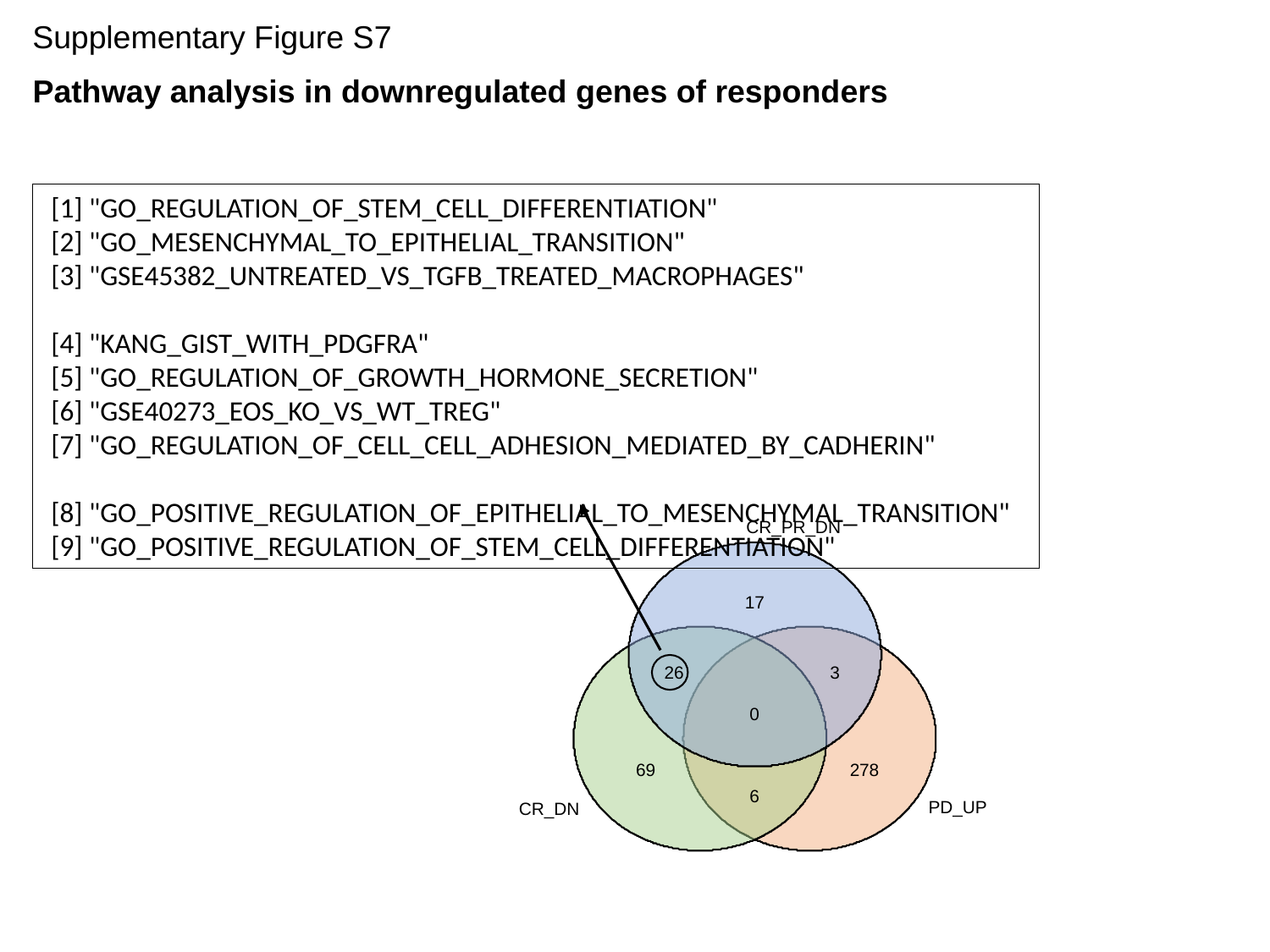

Supplementary Figure S7
Pathway analysis in downregulated genes of responders
 [1] "GO_REGULATION_OF_STEM_CELL_DIFFERENTIATION"
 [2] "GO_MESENCHYMAL_TO_EPITHELIAL_TRANSITION"
 [3] "GSE45382_UNTREATED_VS_TGFB_TREATED_MACROPHAGES"
 [4] "KANG_GIST_WITH_PDGFRA"
 [5] "GO_REGULATION_OF_GROWTH_HORMONE_SECRETION"
 [6] "GSE40273_EOS_KO_VS_WT_TREG"
 [7] "GO_REGULATION_OF_CELL_CELL_ADHESION_MEDIATED_BY_CADHERIN"
 [8] "GO_POSITIVE_REGULATION_OF_EPITHELIAL_TO_MESENCHYMAL_TRANSITION"
 [9] "GO_POSITIVE_REGULATION_OF_STEM_CELL_DIFFERENTIATION"
CR_PR_DN
17
26
3
0
69
278
6
PD_UP
CR_DN
