# Supplementary material for: Combination of gene set signatures correlates with response to nivolumab in platinum-resistant ovarian cancer
Source: Sci Rep. 2021 Jun 1;11:11427. doi: 10.1038/s41598-021-91012-w (PMC8169687; doi:10.1038/s41598-021-91012-w)
Supplement: Supplementary file 14 — Supplementary Information 14. [file 41598_2021_91012_MOESM14_ESM.docx]

**Supplementary Information**

**Supplementary Figure S1**

**Significance testing of ovarian signature scores stratified by histopathological subtype**

Figure interpretation is similar to Figure 1.

The Supplementary Figure S1 image was created using Prism version 8.3.0 (https://www.graphpad.com/).

**Supplementary figure S2**

**Multi-signature heatmap organized by response**

A heatmap representation of all patients and their ovarian-cancer specific scores, similar to Figure 2 but affinely re-scaled per patient and ordered by response group.

The Supplementary Figure S2 image was created using Python 3.0 (https://www.python.org/download/releases/3.0/).

**Supplementary Figure S3**

**Per-patient normalized multi-signature heatmap**

Data is identical to Supplementary Figure S2, but organized by clustering of per-patient normalized scores.

The Supplementary Figure S3 image was created using Python 3.0 (https://www.python.org/download/releases/3.0/).

**Supplemental Figure S4**

**Animation of signature score projection and response**

A 3D multi-view projection of patient signature scores and their clinical responses.

Notation : Clear_cell, ssGSEA score of clear cell gene signature; Proriferative, ssGSEA score of Proliferative gene signature; Immunoreactive, ssGSEA score of Immunoreactive gene signature.

Point colors are CR: green, PR: red, SD: blue, and PD: black.

The Supplementary Figure S4 movie was created using R statistical environment version 3.6.0 (http://www.r-project.org).

**Supplementary Figure S5**

**Distributions and correlations among the five ovarian cancer gene signatures**

Histograms (diagonal), Pearson correlation metrics (upper triangle) and paired scatterplots (lower triangle) of ssGSEA scores are shown. The ssGSEA score of the clear cell gene signature histogram has two peaks, whereas others have dispersed distributions. A negative correlation between the proliferative gene signature score and the clear cell score was observed. A positive correlation between clear cell and differentiated gene signature score was observed. Notation: Dif, Differentiated gene signature score; Imr, Immunoreactive gene signature score; Mes, Mesenchymal gene signature score; Pro, Proliferative gene signature score; Cle,Clear cell gene signature score.

The Supplementary Figure S5 image was created using R statistical environment version 3.6.0 (http://www.r-project.org).

**Supplemental Figure S6**

**Pathway commonality analysis in upregulated genes of responders**

A Venn diagram analysis of statistically significant pathways obtained by samroc analysis, with comparison to significant pathways in downregulated genes of PD patients.

The Supplementary Figure S6 image was created using Python 3.0 (https://www.python.org/download/releases/3.0/).

**Supplementary Figure S7**

**Pathway analysis in downregulated genes of responders**

Similar to Supplementary Figure S6, common pathways are analyzed, though here the comparison is between pathways of downregulated genes in responders versus pathways in upregulated genes in the PD group.

The Supplementary Figure S7 image was created using Python 3.0 (https://www.python.org/download/releases/3.0/).

**Supplementary Table S1**

**Pathways in genes upregulated in CR patients**

Pathway scores are generated by samroc. Columns include samroc-based rank, samroc statistic values, initial p-values, and adjustment of p-values to FDR q-values based on p-value distribution.

**Supplementary Table S2**

**Pathways in genes downregulated in CR patients**

Columns are identical to Supplementary Table S1.

**Supplemental Table S3**

**Pathways in genes upregulated in PD patients**

Columns are identical to Supplementary Table S1.

**Supplementary Table S4**

**Pathways in genes downregulated in PD patients**

Columns are identical to Supplementary Table S1. Multiple immune response pathways are identified.

**Supplementary Table S5**

**Pathways in genes upregulated in CR or PR patients**

Columns are identical to Supplementary Table S1.

**Supplementary Table S6**

**Pathways in genes downregulated in CR or PR patients**

Columns are identical to Supplementary Table S1.
